# (3-Phenyl­sulfanyl-1-phenyl­sulfonyl-1*H*-indol-2-yl)methyl acetate

**DOI:** 10.1107/S1600536811014802

**Published:** 2011-04-29

**Authors:** Alagappa Rammohan, E. Govindan, A. SubbiahPandi, R. Sureshbabu, A. K. Mohana Krishnan

**Affiliations:** a Department of Physics, Presidency College (Autonomous), Chennai 600 005, India; b Department of Organic Chemistry, University of Madras, Guindy Campus, Chennai 600 025, India

## Abstract

In the title compound, C_23_H_19_NO_4_S_2_, the indole ring system makes dihedral angles of 89.6 (1) and 84.5 (8)° with the phenyl­sulfonyl and phenyl­sulfanyl rings, respectively. In the crystal, the mol­ecules are linked into *C*(10) chains running along the *c* axis by an inter­molecular C—H⋯O hydrogen bond. In addition, the crystal packing is stabilized by C—H⋯π inter­actions.

## Related literature

For biological activities of indole derivatives, see: Singh *et al.* (2000 ▶); Andreani *et al.* (2001 ▶); Quetin-Leclercq (1994 ▶); Mukhopadhyay *et al.* (1981 ▶); Taylor *et al.* (1999 ▶); Williams *et al.* (1993 ▶); Sivaraman *et al.* (1996 ▶). For related structures, see: Ravishankar *et al.* (2005 ▶); Chakkaravarthi *et al.* (2008 ▶). For graph-set notation of hydrogen bonds, see: Bernstein *et al.* (1995 ▶).
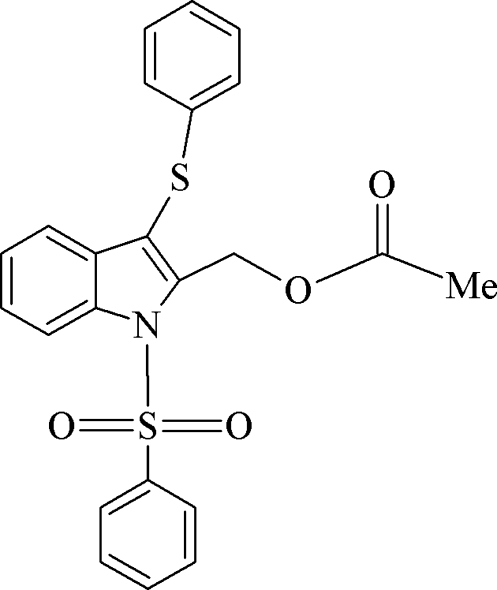


## Experimental

### 

#### Crystal data

C_23_H_19_NO_4_S_2_
*M* *_r_* = 437.51
Monoclinic, 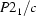

*a* = 14.6530 (6) Å
*b* = 9.4482 (4) Å
*c* = 15.2461 (7) Å
β = 97.055 (3)°
*V* = 2094.76 (16) Å^3^
*Z* = 4
Mo *K*α radiation
μ = 0.29 mm^−1^
*T* = 293 K
0.25 × 0.22 × 0.19 mm

#### Data collection

Bruker APEXII CCD area-detector diffractometer
Absorption correction: multi-scan (*SADABS*; Sheldrick, 1996 ▶) *T*_min_ = 0.981, *T*_max_ = 0.985
19397 measured reflections
5235 independent reflections
3638 reflections with *I* > 2σ(*I*)
*R*_int_ = 0.027

#### Refinement

*R*[*F* ^2^ > 2σ(*F* ^2^)] = 0.039
*wR*(*F* ^2^) = 0.104
*S* = 1.03
5235 reflections
272 parameters
H-atom parameters constrained
Δρ_max_ = 0.26 e Å^−3^
Δρ_min_ = −0.29 e Å^−3^

### 

Data collection: *APEX2* (Bruker, 2004 ▶); cell refinement: *APEX2*; data reduction: *SAINT* (Bruker, 2004 ▶); program(s) used to solve structure: *SHELXS97* (Sheldrick, 2008 ▶); program(s) used to refine structure: *SHELXL97* (Sheldrick, 2008 ▶); molecular graphics: *ORTEP-3* (Farrugia, 1997 ▶); software used to prepare material for publication: *SHELXL97* and *PLATON* (Spek, 2009 ▶).

## Supplementary Material

Crystal structure: contains datablocks global, I. DOI: 10.1107/S1600536811014802/bt5514sup1.cif

Structure factors: contains datablocks I. DOI: 10.1107/S1600536811014802/bt5514Isup2.hkl

Supplementary material file. DOI: 10.1107/S1600536811014802/bt5514Isup3.cml

Additional supplementary materials:  crystallographic information; 3D view; checkCIF report

## Figures and Tables

**Table 1:** Hydrogen-bond geometry (Å, °) *Cg*1 and *Cg*2 are the centroids of the N1/C1/C6–C8 and C1–C6 rings, respectively.

*D*—H⋯*A*	*D*—H	H⋯*A*	*D*⋯*A*	*D*—H⋯*A*
C4—H4⋯O4^i^	0.93	2.59	3.274 (2)	131
C15—H15⋯*Cg*1^ii^	0.93	2.77	3.559 (2)	143
C16—H16⋯*Cg*2^ii^	0.93	2.72	3.5146 (19)	143

Symmetry codes: (i) 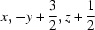; (ii) 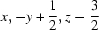.

**Fig. 1.**
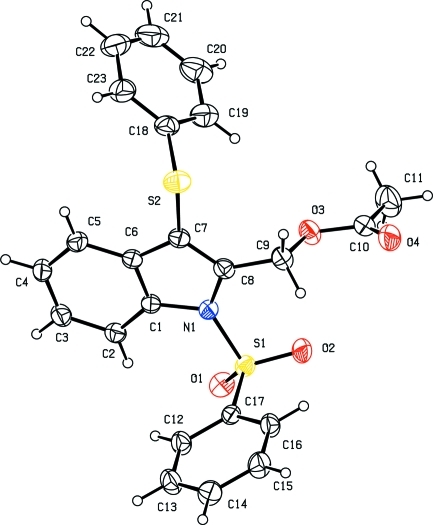
View of the title molecule with the atom labeling scheme. The displacement ellipsoids are drawn at the 30% probability level while the H atoms are shown as small spheres of arbitrary radii.

**Fig. 2.**
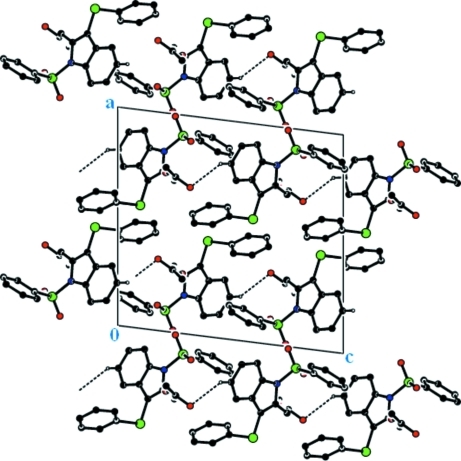
The molecular packing viewed down the b axis.

## supplementary crystallographic information

### Comment

Indole derivatives have been found to exhibit antibacterial, antifungal (Singh *et al.*, 2000) and antitumour activities (Andreani *et al.*, 2001). Some of the indole alkaloids extracted from plants possess interesting cytotoxic, antitumour or antiparasitic properties (Quetin-Leclercq, 1994; Mukhopadhyay *et al.*, 1981). Pyrido[1,2-*a*]indole derivatives have been identified as potent inhibitors of human immunodeficiency virus type 1 (Taylor *et al.*, 1999), and 5-chloro-3-(phenylsulfonyl)indole-2-carboxamide is reported to be a highly potent non-nucleoside inhibitor of HIV-1 reverse transcriptase (Williams *et al.*,1993). The interaction of phenylsulfonylindole with calf thymus DNA has also been studied by spectroscopic methods (Sivaraman *et al.*, 1996). Against this background, and in order to obtain detailed information on molecular conformations in the solid state, X-ray studies of the title compound (I) have been carried out.

X-Ray analysis confirms the molecular structure and atom connectivity for (I), as illustrated in Fig. 1. The indole ring system is essentially planar, with maximum deviation of 0.020 (2) Å for atom N1. The mean planes of the indole ring system make a dihedral algles of 89.6 (1) and 84.5 (8)° with respect to the phenyl rings, it shows that both the phenyl rings are perpendicular with respect to the indole ring system. The S—O, S—C, and S—N distances are 1.420 (12), 1.754 (17) and 1.676 (14) Å, respectively, these are comparable as observed in similar structures (Ravishankar *et al.*, 2005). As a result of the electron-withdrawing character of the phenylsulfonyl group, the N—C*sp*^2^ bond lengths, *viz*. N1—C1 [1.422 (2) Å] and N1—C8 [1.418 (2) Å], are longer than the mean value of 1.355 (14) Å reported for N atoms with planar configurations.

The S atom exhibits significant deviation from that of a regular tetrahedron, with the largest deviations being seen for the O—S—O [O1—S1—O2 120.3 (7)°] and O—S—N angles [O1—S1—N1 105.4 (7)°]. The widening of the angles may be due to repulsive interactions between the two short S═O bonds, similar to what is observed in related structures (Chakkaravarthi *et al.*, 2008). The atom C4 act as a donor to the atom O4 of the neighbouring molecule at (*x*, 3/2 - *y*, 1/2 + *z*). This hydrogen bond is involved in a motif C(10) chain along *b* axis. In addition to van der Waals interaction, the crystal packing is stabilized by C—H..O and C—H···π interactions.

### Experimental

To solution of 2-(bromomethyl)-1-(phenyl sulfonyl)-3-(phenylthio)-1*H*-indole (2.18 mmol) in dry dimethyl formamide (10 ml), potassium acetate (4.36 mmol) was added under nitrogen atmosphere, the reaction mixture was stirred at room temperature for 5 h, then it was poured over crushed ice (50 g) containing 1 ml of concentrated hydrochloric acid. The obtained brown solid was filtered and dried. Single crystals of the title compound suitable for X-ray diffraction were obtained by slow evaporation of a solution in methanol.

### Refinement

All H atoms were fixed geometrically and allowed to ride on their parent C atoms, with C—H distances fixed in the range 0.93–0.97 Å with *U*_iso_(H) = 1.5*U*_eq_(C) for methyl H 1.2*U*_eq_(C) for other H atoms.

### Crystal data

**Table:**

C_23_H_19_NO_4_S_2_	*F*(000) = 912
*M**_r_* = 437.51	*D*_x_ = 1.387 Mg m^−^^3^
Monoclinic, *P*2_1_/*c*	Mo *K*α radiation, λ = 0.71073 Å
Hall symbol: -P 2ybc	Cell parameters from 5235 reflections
*a* = 14.6530 (6) Å	θ = 1.4–28.4°
*b* = 9.4482 (4) Å	µ = 0.29 mm^−^^1^
*c* = 15.2461 (7) Å	*T* = 293 K
β = 97.055 (3)°	Block, white
*V* = 2094.76 (16) Å^3^	0.25 × 0.22 × 0.19 mm
*Z* = 4	

### Data collection

**Table:**

Bruker APEXII CCD area-detector diffractometer	5235 independent reflections
Radiation source: fine-focus sealed tube	3638 reflections with *I* > 2σ(*I*)
graphite	*R*_int_ = 0.027
ω and φ scans	θ_max_ = 28.4°, θ_min_ = 1.4°
Absorption correction: multi-scan (*SADABS*; Sheldrick, 1996)	*h* = −19→19
*T*_min_ = 0.981, *T*_max_ = 0.985	*k* = −11→12
19397 measured reflections	*l* = −20→20

### Refinement

**Table:**

Refinement on *F*^2^	Primary atom site location: structure-invariant direct methods
Least-squares matrix: full	Secondary atom site location: difference Fourier map
*R*[*F*^2^ > 2σ(*F*^2^)] = 0.039	Hydrogen site location: inferred from neighbouring sites
*wR*(*F*^2^) = 0.104	H-atom parameters constrained
*S* = 1.03	*w* = 1/[σ^2^(*F*_o_^2^) + (0.0433*P*)^2^ + 0.4791*P*] where *P* = (*F*_o_^2^ + 2*F*_c_^2^)/3
5235 reflections	(Δ/σ)_max_ < 0.001
272 parameters	Δρ_max_ = 0.26 e Å^−^^3^
0 restraints	Δρ_min_ = −0.29 e Å^−^^3^

### Special details

**Table:**

Geometry. All e.s.d.'s (except the e.s.d. in the dihedral angle between two l.s. planes) are estimated using the full covariance matrix. The cell e.s.d.'s are taken into account individually in the estimation of e.s.d.'s in distances, angles and torsion angles; correlations between e.s.d.'s in cell parameters are only used when they are defined by crystal symmetry. An approximate (isotropic) treatment of cell e.s.d.'s is used for estimating e.s.d.'s involving l.s. planes.
Refinement. Refinement of *F*^2^ against ALL reflections. The weighted *R*-factor *wR* and goodness of fit *S* are based on *F*^2^, conventional *R*-factors *R* are based on *F*, with *F* set to zero for negative *F*^2^. The threshold expression of *F*^2^ > σ(*F*^2^) is used only for calculating *R*-factors(gt) *etc*. and is not relevant to the choice of reflections for refinement. *R*-factors based on *F*^2^ are statistically about twice as large as those based on *F*, and *R*-factors based on ALL data will be even larger.

### Fractional atomic coordinates and isotropic or equivalent isotropic displacement parameters (Å^2^)

**Table:**

	*x*	*y*	*z*	*U*_iso_*/*U*_eq_	
C1	0.16888 (11)	0.62556 (16)	0.35882 (10)	0.0387 (4)	
C2	0.09311 (12)	0.55131 (18)	0.38072 (11)	0.0463 (4)	
H2	0.0349	0.5645	0.3500	0.056*	
C3	0.10805 (13)	0.45722 (19)	0.44998 (12)	0.0530 (4)	
H3	0.0586	0.4057	0.4660	0.064*	
C4	0.19466 (14)	0.43671 (19)	0.49671 (12)	0.0543 (5)	
H4	0.2019	0.3728	0.5434	0.065*	
C5	0.26938 (13)	0.50947 (18)	0.47475 (11)	0.0482 (4)	
H5	0.3273	0.4959	0.5061	0.058*	
C6	0.25666 (11)	0.60461 (16)	0.40419 (10)	0.0410 (4)	
C7	0.31844 (11)	0.69850 (17)	0.36612 (11)	0.0424 (4)	
C8	0.27026 (11)	0.77336 (17)	0.30037 (11)	0.0422 (4)	
C9	0.30417 (12)	0.89164 (18)	0.24908 (12)	0.0494 (4)	
H9A	0.3708	0.8898	0.2536	0.059*	
H9B	0.2795	0.8842	0.1872	0.059*	
C10	0.28751 (13)	1.14037 (19)	0.24295 (13)	0.0534 (4)	
C11	0.2490 (2)	1.2651 (2)	0.28528 (19)	0.0882 (8)	
H11A	0.2619	1.3495	0.2540	0.132*	
H11B	0.2765	1.2722	0.3456	0.132*	
H11C	0.1837	1.2541	0.2836	0.132*	
C12	0.07661 (13)	0.49985 (18)	0.14300 (12)	0.0522 (4)	
H12	0.0490	0.4775	0.1930	0.063*	
C13	0.08604 (15)	0.3989 (2)	0.08013 (13)	0.0622 (5)	
H13	0.0656	0.3071	0.0880	0.075*	
C14	0.12545 (14)	0.4326 (2)	0.00578 (13)	0.0600 (5)	
H14	0.1311	0.3637	−0.0368	0.072*	
C15	0.15663 (14)	0.5672 (2)	−0.00607 (12)	0.0598 (5)	
H15	0.1831	0.5894	−0.0567	0.072*	
C16	0.14880 (12)	0.66998 (19)	0.05698 (11)	0.0506 (4)	
H16	0.1703	0.7612	0.0495	0.061*	
C17	0.10864 (10)	0.63531 (16)	0.13106 (10)	0.0391 (3)	
C18	0.43670 (12)	0.7939 (2)	0.50554 (13)	0.0557 (5)	
C19	0.39555 (14)	0.9229 (3)	0.51322 (15)	0.0681 (6)	
H19	0.3656	0.9675	0.4634	0.082*	
C20	0.39877 (17)	0.9870 (3)	0.59577 (18)	0.0861 (8)	
H20	0.3692	1.0730	0.6018	0.103*	
C21	0.4459 (2)	0.9222 (4)	0.66824 (17)	0.0938 (9)	
H21	0.4489	0.9654	0.7233	0.113*	
C22	0.48819 (18)	0.7960 (4)	0.66036 (17)	0.0896 (8)	
H22	0.5203	0.7537	0.7099	0.108*	
C23	0.48383 (15)	0.7297 (3)	0.57906 (15)	0.0736 (6)	
H23	0.5123	0.6426	0.5739	0.088*	
N1	0.17660 (9)	0.73225 (14)	0.29437 (9)	0.0405 (3)	
O1	0.01013 (8)	0.74327 (13)	0.24414 (9)	0.0550 (3)	
O2	0.11545 (9)	0.90024 (12)	0.17575 (8)	0.0561 (3)	
O3	0.27314 (8)	1.02147 (12)	0.28681 (8)	0.0544 (3)	
O4	0.32709 (11)	1.14258 (15)	0.17888 (9)	0.0712 (4)	
S1	0.09482 (3)	0.76585 (4)	0.21008 (3)	0.04212 (12)	
S2	0.43695 (3)	0.71174 (6)	0.40011 (3)	0.05871 (15)	

### Atomic displacement parameters (Å^2^)

**Table:**

	*U*^11^	*U*^22^	*U*^33^	*U*^12^	*U*^13^	*U*^23^
C1	0.0493 (9)	0.0344 (8)	0.0324 (8)	−0.0008 (7)	0.0050 (7)	−0.0040 (7)
C2	0.0514 (10)	0.0450 (9)	0.0421 (10)	−0.0070 (8)	0.0040 (8)	−0.0032 (8)
C3	0.0662 (12)	0.0463 (10)	0.0473 (11)	−0.0130 (9)	0.0105 (9)	0.0007 (8)
C4	0.0806 (13)	0.0408 (9)	0.0405 (10)	−0.0069 (9)	0.0033 (9)	0.0048 (8)
C5	0.0604 (11)	0.0417 (9)	0.0404 (9)	0.0018 (8)	−0.0027 (8)	−0.0027 (8)
C6	0.0519 (9)	0.0353 (8)	0.0355 (9)	0.0005 (7)	0.0036 (7)	−0.0066 (7)
C7	0.0454 (9)	0.0422 (9)	0.0397 (9)	−0.0008 (7)	0.0057 (7)	−0.0065 (7)
C8	0.0481 (9)	0.0408 (9)	0.0390 (9)	−0.0030 (7)	0.0105 (7)	−0.0067 (7)
C9	0.0580 (10)	0.0438 (9)	0.0487 (10)	−0.0043 (8)	0.0162 (8)	−0.0032 (8)
C10	0.0585 (11)	0.0451 (10)	0.0539 (12)	−0.0092 (8)	−0.0044 (9)	0.0015 (9)
C11	0.113 (2)	0.0501 (13)	0.102 (2)	0.0067 (12)	0.0184 (16)	−0.0035 (13)
C12	0.0707 (12)	0.0442 (10)	0.0436 (10)	−0.0074 (8)	0.0148 (9)	0.0013 (8)
C13	0.0913 (15)	0.0389 (10)	0.0581 (12)	−0.0086 (10)	0.0158 (11)	−0.0035 (9)
C14	0.0825 (14)	0.0500 (11)	0.0482 (11)	0.0101 (10)	0.0100 (10)	−0.0079 (9)
C15	0.0806 (13)	0.0579 (12)	0.0446 (11)	0.0002 (10)	0.0220 (10)	0.0007 (9)
C16	0.0657 (11)	0.0422 (9)	0.0455 (10)	−0.0056 (8)	0.0128 (9)	0.0043 (8)
C17	0.0436 (8)	0.0376 (8)	0.0354 (9)	0.0030 (7)	0.0015 (7)	0.0017 (7)
C18	0.0447 (10)	0.0705 (13)	0.0511 (11)	−0.0171 (9)	0.0025 (8)	−0.0018 (10)
C19	0.0653 (13)	0.0789 (15)	0.0596 (13)	−0.0093 (11)	0.0061 (10)	−0.0100 (11)
C20	0.0887 (17)	0.0921 (18)	0.0806 (18)	−0.0225 (14)	0.0230 (14)	−0.0285 (15)
C21	0.101 (2)	0.126 (3)	0.0559 (15)	−0.0570 (19)	0.0151 (14)	−0.0210 (17)
C22	0.0854 (18)	0.126 (2)	0.0537 (15)	−0.0416 (17)	−0.0083 (12)	0.0084 (16)
C23	0.0645 (13)	0.0873 (16)	0.0653 (15)	−0.0191 (11)	−0.0060 (11)	0.0076 (13)
N1	0.0465 (7)	0.0395 (7)	0.0350 (7)	−0.0020 (6)	0.0034 (6)	0.0009 (6)
O1	0.0473 (7)	0.0625 (8)	0.0560 (8)	0.0126 (6)	0.0097 (6)	−0.0015 (6)
O2	0.0733 (8)	0.0355 (6)	0.0579 (8)	0.0073 (6)	0.0010 (6)	0.0053 (6)
O3	0.0705 (8)	0.0421 (7)	0.0537 (7)	−0.0073 (6)	0.0205 (6)	−0.0047 (6)
O4	0.0962 (11)	0.0635 (9)	0.0545 (9)	−0.0183 (8)	0.0113 (8)	0.0085 (7)
S1	0.0477 (2)	0.0375 (2)	0.0408 (2)	0.00722 (17)	0.00382 (18)	0.00013 (17)
S2	0.0446 (3)	0.0741 (3)	0.0573 (3)	−0.0010 (2)	0.0058 (2)	−0.0084 (3)

### Geometric parameters (Å, °)

**Table:**

C1—C2	1.388 (2)	C12—C17	1.383 (2)
C1—C6	1.398 (2)	C12—H12	0.9300
C1—N1	1.422 (2)	C13—C14	1.371 (3)
C2—C3	1.377 (2)	C13—H13	0.9300
C2—H2	0.9300	C14—C15	1.371 (3)
C3—C4	1.391 (3)	C14—H14	0.9300
C3—H3	0.9300	C15—C16	1.381 (3)
C4—C5	1.369 (2)	C15—H15	0.9300
C4—H4	0.9300	C16—C17	1.376 (2)
C5—C6	1.397 (2)	C16—H16	0.9300
C5—H5	0.9300	C17—S1	1.7532 (16)
C6—C7	1.440 (2)	C18—C19	1.371 (3)
C7—C8	1.353 (2)	C18—C23	1.382 (3)
C7—S2	1.7545 (17)	C18—S2	1.785 (2)
C8—N1	1.418 (2)	C19—C20	1.392 (3)
C8—C9	1.484 (2)	C19—H19	0.9300
C9—O3	1.451 (2)	C20—C21	1.373 (4)
C9—H9A	0.9700	C20—H20	0.9300
C9—H9B	0.9700	C21—C22	1.356 (4)
C10—O4	1.196 (2)	C21—H21	0.9300
C10—O3	1.337 (2)	C22—C23	1.383 (4)
C10—C11	1.488 (3)	C22—H22	0.9300
C11—H11A	0.9600	C23—H23	0.9300
C11—H11B	0.9600	N1—S1	1.6763 (14)
C11—H11C	0.9600	O1—S1	1.4190 (12)
C12—C13	1.371 (3)	O2—S1	1.4201 (12)
			
C2—C1—C6	121.60 (15)	C12—C13—H13	119.8
C2—C1—N1	131.17 (15)	C14—C13—H13	119.8
C6—C1—N1	107.21 (13)	C15—C14—C13	120.33 (18)
C3—C2—C1	117.01 (17)	C15—C14—H14	119.8
C3—C2—H2	121.5	C13—C14—H14	119.8
C1—C2—H2	121.5	C14—C15—C16	120.22 (17)
C2—C3—C4	122.14 (17)	C14—C15—H15	119.9
C2—C3—H3	118.9	C16—C15—H15	119.9
C4—C3—H3	118.9	C17—C16—C15	118.97 (16)
C5—C4—C3	120.78 (17)	C17—C16—H16	120.5
C5—C4—H4	119.6	C15—C16—H16	120.5
C3—C4—H4	119.6	C16—C17—C12	120.98 (15)
C4—C5—C6	118.46 (17)	C16—C17—S1	119.59 (13)
C4—C5—H5	120.8	C12—C17—S1	119.40 (12)
C6—C5—H5	120.8	C19—C18—C23	120.1 (2)
C5—C6—C1	120.00 (15)	C19—C18—S2	120.85 (16)
C5—C6—C7	132.54 (16)	C23—C18—S2	118.90 (18)
C1—C6—C7	107.41 (14)	C18—C19—C20	119.8 (2)
C8—C7—C6	108.92 (14)	C18—C19—H19	120.1
C8—C7—S2	126.09 (13)	C20—C19—H19	120.1
C6—C7—S2	124.99 (13)	C21—C20—C19	119.4 (3)
C7—C8—N1	108.51 (14)	C21—C20—H20	120.3
C7—C8—C9	127.31 (15)	C19—C20—H20	120.3
N1—C8—C9	123.79 (15)	C22—C21—C20	120.7 (3)
O3—C9—C8	106.62 (12)	C22—C21—H21	119.6
O3—C9—H9A	110.4	C20—C21—H21	119.6
C8—C9—H9A	110.4	C21—C22—C23	120.4 (3)
O3—C9—H9B	110.4	C21—C22—H22	119.8
C8—C9—H9B	110.4	C23—C22—H22	119.8
H9A—C9—H9B	108.6	C18—C23—C22	119.4 (3)
O4—C10—O3	123.03 (18)	C18—C23—H23	120.3
O4—C10—C11	126.05 (19)	C22—C23—H23	120.3
O3—C10—C11	110.92 (18)	C8—N1—C1	107.94 (13)
C10—C11—H11A	109.5	C8—N1—S1	126.33 (11)
C10—C11—H11B	109.5	C1—N1—S1	123.64 (11)
H11A—C11—H11B	109.5	C10—O3—C9	115.82 (13)
C10—C11—H11C	109.5	O1—S1—O2	120.31 (7)
H11A—C11—H11C	109.5	O1—S1—N1	105.42 (7)
H11B—C11—H11C	109.5	O2—S1—N1	106.70 (7)
C13—C12—C17	119.13 (16)	O1—S1—C17	109.01 (8)
C13—C12—H12	120.4	O2—S1—C17	109.15 (8)
C17—C12—H12	120.4	N1—S1—C17	105.16 (7)
C12—C13—C14	120.36 (17)	C7—S2—C18	100.69 (8)
			
C6—C1—C2—C3	0.8 (2)	C19—C20—C21—C22	−0.9 (4)
N1—C1—C2—C3	−177.37 (16)	C20—C21—C22—C23	−0.5 (4)
C1—C2—C3—C4	0.2 (3)	C19—C18—C23—C22	0.7 (3)
C2—C3—C4—C5	−0.6 (3)	S2—C18—C23—C22	176.63 (16)
C3—C4—C5—C6	−0.1 (3)	C21—C22—C23—C18	0.6 (3)
C4—C5—C6—C1	1.1 (2)	C7—C8—N1—C1	−1.09 (17)
C4—C5—C6—C7	178.16 (17)	C9—C8—N1—C1	−174.33 (14)
C2—C1—C6—C5	−1.5 (2)	C7—C8—N1—S1	−165.03 (11)
N1—C1—C6—C5	177.06 (14)	C9—C8—N1—S1	21.7 (2)
C2—C1—C6—C7	−179.21 (14)	C2—C1—N1—C8	179.43 (16)
N1—C1—C6—C7	−0.66 (16)	C6—C1—N1—C8	1.06 (16)
C5—C6—C7—C8	−177.32 (17)	C2—C1—N1—S1	−16.1 (2)
C1—C6—C7—C8	−0.01 (18)	C6—C1—N1—S1	165.54 (11)
C5—C6—C7—S2	2.9 (3)	O4—C10—O3—C9	−3.4 (3)
C1—C6—C7—S2	−179.79 (12)	C11—C10—O3—C9	177.29 (17)
C6—C7—C8—N1	0.68 (18)	C8—C9—O3—C10	−172.38 (15)
S2—C7—C8—N1	−179.54 (11)	C8—N1—S1—O1	−163.03 (13)
C6—C7—C8—C9	173.61 (15)	C1—N1—S1—O1	35.40 (14)
S2—C7—C8—C9	−6.6 (2)	C8—N1—S1—O2	−34.03 (15)
C7—C8—C9—O3	−100.22 (19)	C1—N1—S1—O2	164.40 (12)
N1—C8—C9—O3	71.71 (19)	C8—N1—S1—C17	81.83 (14)
C17—C12—C13—C14	−1.0 (3)	C1—N1—S1—C17	−79.75 (13)
C12—C13—C14—C15	0.6 (3)	C16—C17—S1—O1	143.04 (14)
C13—C14—C15—C16	0.2 (3)	C12—C17—S1—O1	−35.16 (16)
C14—C15—C16—C17	−0.6 (3)	C16—C17—S1—O2	9.83 (16)
C15—C16—C17—C12	0.2 (3)	C12—C17—S1—O2	−168.37 (14)
C15—C16—C17—S1	−177.98 (14)	C16—C17—S1—N1	−104.33 (14)
C13—C12—C17—C16	0.6 (3)	C12—C17—S1—N1	77.48 (15)
C13—C12—C17—S1	178.76 (15)	C8—C7—S2—C18	110.04 (16)
C23—C18—C19—C20	−2.1 (3)	C6—C7—S2—C18	−70.22 (16)
S2—C18—C19—C20	−177.95 (16)	C19—C18—S2—C7	−58.87 (17)
C18—C19—C20—C21	2.2 (3)	C23—C18—S2—C7	125.22 (16)

### Hydrogen-bond geometry (Å, °)

**Table:**

Cg1 and Cg2 are the centroids of the N1/C1/C6–C8 and C1–C6 rings, respectively.

**Table:**

*D*—H···*A*	*D*—H	H···*A*	*D*···*A*	*D*—H···*A*
C4—H4···O4^i^	0.93	2.59	3.274 (2)	131.
C15—H15···Cg1^ii^	0.93	2.77	3.559 (2)	143
C16—H16···Cg2^ii^	0.93	2.72	3.5146 (19)	143

Symmetry codes: (i) *x*, −*y*+3/2, *z*+1/2; (ii) *x*, −*y*+1/2, *z*−3/2.
